# Upregulation of Autophagy During the Differentiation of Primary Human Term Cytotrophoblast Cells into Syncytial Cells: Ultrastructural Analysis

**DOI:** 10.3390/ijms26031321

**Published:** 2025-02-04

**Authors:** Shohei Tozawa, Hironori Takahashi, Syunya Noguchi, Takami Takizawa, Takanobu Sakurai, Akihide Ohkuchi, Hiroyuki Fujiwara, Toshihiro Takizawa

**Affiliations:** 1 Department of Molecular Medicine and Anatomy, Nippon Medical School, Tokyo 113-8602, Japan; shohei-tozawa@nms.ac.jp (S.T.); n-syunya@nms.ac.jp (S.N.); takamit@nms.ac.jp (T.T.); t-sakurai@nms.ac.jp (T.S.); 2 Department of Obstetrics and Gynecology, Jichi Medical University, Tochigi 329-0498, Japan; hironori@jichi.ac.jp (H.T.); okuchi@jichi.ac.jp (A.O.); fujiwara@jichi.ac.jp (H.F.)

**Keywords:** human term placenta, villous trophoblast, cytotrophoblast, syncytiotrophoblast, primary culture, autophagy, electron microscopy

## Abstract

The villous trophoblast cells are of fundamental importance because they fulfill a variety of functions that are vital for the growth of the fetus and the maintenance of pregnancy. A simple in vitro villous trophoblast cell model that grows on standard tissue culture plates has been utilized for various functional studies on villous trophoblast cells. Despite the potential value of incorporating electron microscopy analysis in reports on functional analysis of primary human trophoblast cells, electron microscopy analysis is exclusively ancillary to functional analysis in previous publications. In the context of autophagy research of villous trophoblast cells using primary trophoblast cells, a detailed ultrastructural analysis of autophagy flux using electron microscopy is imperative; however, it has not been conducted to date. In this study, we isolated term villous trophoblast cells (i.e., cytotrophoblast cells, CTB cells) using the most up-to-date isolation method for isolating pure CTB cells from human term placenta and investigated the ultrastructural dynamic process of autophagy of cultured CTB cells by means of transmission electron microscopy. The initial 6 h culture resulted in CTB cell aggregation; however, the majority of CTB cells did not differentiate into syncytial cells. In contrast, after 72 h, CTB cells exhibited a promotion of differentiation into syncytial cells. The electron microscopy analysis revealed the upregulation of autophagy and visualized unique autophagic profiles during differentiation into syncytial cells, which exhibited perinuclear accumulation of extremely large autophagosomes/autolysosomes. This study provides novel insights into the reproductive biology of primary trophoblast cells, thereby demonstrating the substantial value of primary trophoblast cells as research resources.

## 1. Introduction

The human placenta, comprised of the maternal decidua and the fetal chorion, is a pregnancy-limited organ with diverse functions in fetal development [1]. Chorionic villi, which originate from the chorionic plate, are covered by villous trophoblast cells. These cells have two main types: syncytiotrophoblast (STB) and cytotrophoblast (CTB) [2]. The STB, a fully differentiated multinucleated syncytium, is responsible for covering the villous surface and interacting with maternal blood in the intervillous space. The mononuclear CTB cells, situated beneath the STB, form the inner trophoblast layer. The CTB cells function as progenitor cells for the STB, continuously proliferating, differentiating, and fusing to maintain the post-mitotic STB. The STB plays a pivotal role in orchestrating crucial processes such as fetomaternal exchange and transport, including the transfer of gases, nutrients, waste, and maternal IgG [3]. The STB endocrine function involves the production and secretion of pregnancy-specific hormones and growth factors into the maternal circulation [4]. Moreover, the STB functions as a protective immunological barrier, preventing maternal immune cells from attacking the semi-allograft fetus while shielding the fetus against pathogens [5,6,7]. The STB secretes extracellular vesicles, including exosomes, which have the potential to act as mediators of placenta–maternal cell communication [8]. As described above, the STB is of paramount importance because it fulfills a variety of functions that are vital for the growth of the fetus and the maintenance of pregnancy.

Although the use of ex vivo placenta explants is possible, it is not feasible to conduct in vivo villous trophoblast cell analysis using human placentas due to ethical considerations. Consequently, in vitro analysis of trophoblast cells isolated from human placentas is necessary to better understand the diverse functions of villous trophoblast cells under physiological pathophysiological conditions. However, the study of trophoblast cells, particularly STB, has been hindered by its minimal proliferative capacity, which is characterized as post-mitotic [9,10]. The in vitro formation of STB can be effectively modeled using the human term placenta [11]. A simple in vitro villous trophoblast cell model (i.e., a traditional model) that grows on standard tissue culture plates has been utilized for various functional studies on the STB. These studies have included research on autophagy [12,13,14], innate immune responses [15], drug resistance [16], metabolism [17], placenta-specific microRNAs [18], and STB-derived extracellular vesicles [12]. The majority of these studies have focused on the analysis of cultured cells from the perspectives of both functional analysis with/without light microscopy analysis. Despite the potential value of incorporating electron microscopy analysis in reports on functional analysis of primary human trophoblast cells, electron microscopy analysis is exclusively ancillary to functional analysis in previous publications. In the context of autophagy research of villous trophoblast cells using primary trophoblast cells, a detailed ultrastructural analysis of autophagy flux using electron microscopy is imperative; however, it has not been conducted to date. Therefore, there is a dearth of in-depth analysis of the ultrastructure of the cells using electron microscopy.

In this study, we isolated villous trophoblast cells using the most up-to-date isolation method for isolating pure CTB cells from human term placenta [11] and investigated the ultrastructural dynamic process of autophagy of cultured CTB cells by means of transmission electron microscopy. We found distinctive autophagy profiles subsequent to differentiation into syncytial cells.

## 2. Results

### 2.1. Confocal Microscopy Analysis of CDH1 and KRT7

**Figure 1:**
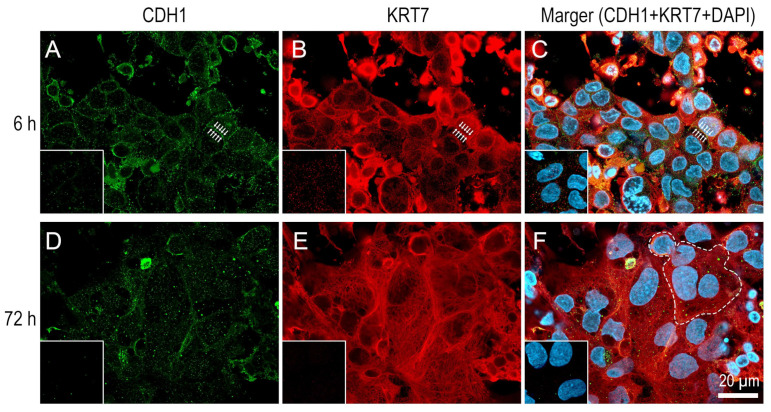
Confocal microscopy analysis of CDH1 and KRT7 in cultured primary human term cytotrophoblast (CTB) cells. (**A**) The subcellular localization of CDH1 (green) in CTB cells at 6 h culture. (**B**) The KRT7 (red) image of cells is shown in A. (**C**) Merged DCH1, KRT7, and DAPI (blue) images of the cells are shown in A. Note that the cells are aggregated; however, the presence of syncytial cells is rarely observed. Intercellular dot-like fluorescence signals at cell–cell contact sites are indicated with arrows. (**D**) The subcellular localization of CDH1 in CTB cells at 72 h culture. CTB cells are fused to form multinucleated syncytial cells. (**E**) The KRT7 image of cells is shown in D. (**F**) Merged DCH1, KRT7, and DAPI images of the cells are shown in D. The delineation of a syncytial cell is represented by a white dotted line. Insets show controls using non-immune IgG. Images are shown at the same magnification.

Confocal microscopy analysis showed dynamic changes in the subcellular localization of cadherin 1 (CDH1, also known as E-cadherin) in isolated CTB cells during differentiation into aggregates and syncytial cells (Figure 1). Mononuclear CTB cells that were cultured for 6 h after isolation already formed aggregates and showed intercellular dot-like fluorescence signals at cell–cell contact sites (Figure 1A,C). As CTB cells were fused to form multinucleated syncytial cells for 72 h, fluorescence signals indicating CDH1 were reduced, being still present at cell–cell contact sites between unfused mononuclear CTB cells and syncytial cells (Figure 1D,F). Within the syncytial cells, irregular punctate localization of CDH1 was also detectable (Figure 1D,F). Controls using non-immune IgG were negative (the insets of Figure 1).

Confocal microscopy analysis for keratin 7 (KRT7, also known as cytokeratin 7) revealed the presence of a network of keratin intermediate filaments extending throughout the cytoplasm of cultured CTB cells (Figure 1B,E). In both 6 and 72 h cultures, KRT7 expression was detected in the vast majority of the cells, if not in all, indicating that pure primary CTB cells were isolated from human term placentas. A subset of KRT7-positive cells showed features such as cytoplasmic shrinkage, condensation, and pyknosis of the nucleus, indicating the presence of apoptosis.

### 2.2. Transmission Electron Microscopy Analysis of Cultured Primary CTB Cells

**Figure 2:**
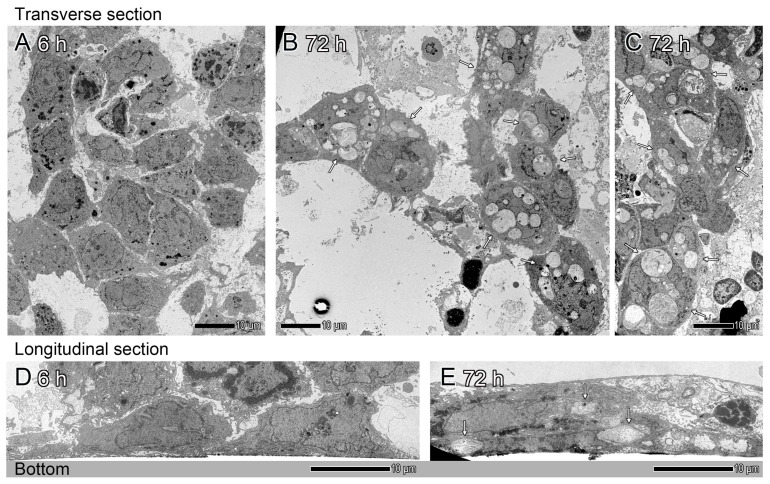
Electron microscopy analysis of cultured primary human term CTB cells. Images of transverse (**A**–**C**) and longitudinal (**D**,**E**) sections of the cultured cells. At 6 h culture, CTB cells that have a cuboidal and polygonal shape are aggregated; however, the presence of syncytial cells is rarely observed (**A**,**D**). At 72 h culture, CTB cells differentiate into multinucleated syncytial cells (**B**,**C**,**E**). Note the presence of a remarkable number of gigantic autophagosomes/autolysosomes (arrows) in the perinuclear area (**B**,**C**,**E**).

**Figure 3:**
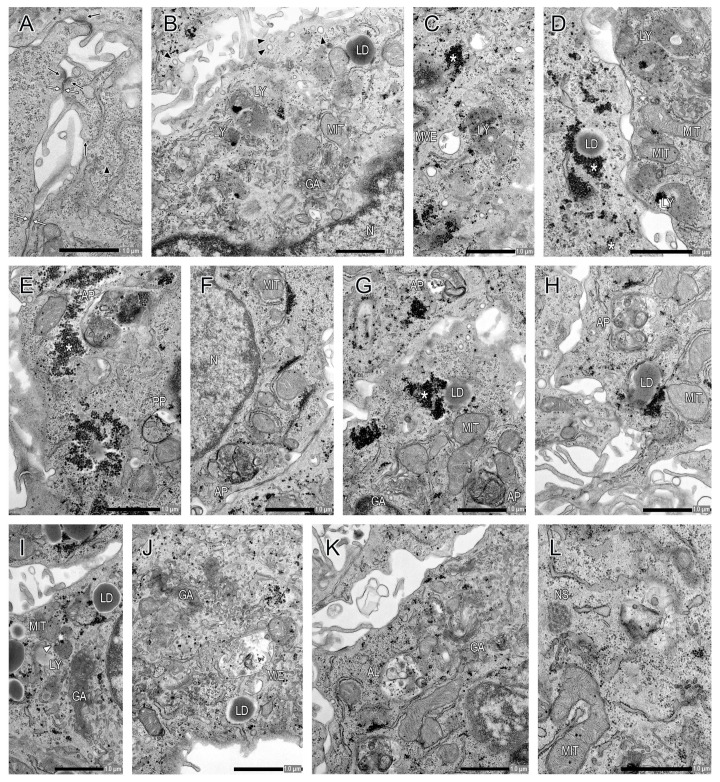
Ultrastructure of primary CTB cells at 6 h culture. (**A**) Desmosomes (black arrows) and primitive adherens junction-like structures (white arrows) are visible at the cell–cell interface. (**B**) Microvilli with coated pits and vesicles (black arrowheads) are evident on the cell surface. (**B**–**K**) In CTB cells, the cytoplasm contains rough endoplasmic reticulum, mitochondria (MIT), free ribosomes, polyribosomes, Golgi apparatus (GA), lysosomes (LY), endosomes that contain multivesicular endosomes (MVEs), lipid droplets (LD), and cytoskeletal filaments, but very few secretory granules. Aggregates of glycogen (*) are often present (**C**–**E**,**G**). The main compartments of the multi-step process of autophagy are detectable: phagophore (PP), autophagosome (AP), and autolysosome (AL) (**E**–**H**,**K**). A mitochondria-derived vesicle is engulfed in a lysosome by invagination of the lysosomal membrane (white arrowhead) (**I**). The compartment that cannot be distinguished as either MVE or endolysosome is denoted as M/E (**J**). (**L**) Nematosome (NS) is seen.

**Figure 4:**
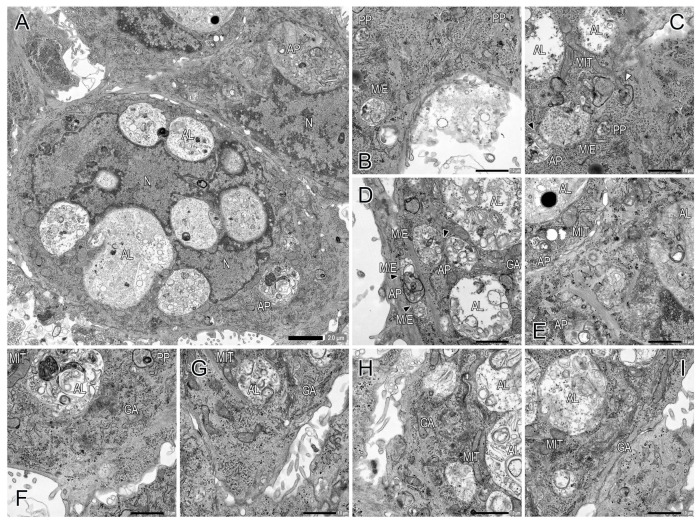
Ultrastructure of primary CTB cells at 72 h culture (part 1). (**A**) CTB cells undergo differentiation into syncytial cells, exhibiting a substantial number of gigantic autophagosomes and autolysosomes in the perinuclear area. Note an increase in the number and size of autophagosomes/autolysosomes. (**B**–**I**) The main compartments of the multi-step process of autophagy are readily detectable: phagophore (PP), autophagosome (AP), and autolysosome (AL). The compartment that cannot be distinguished as either MVE or endolysosome is denoted as M/E. The fusion of MVEs/endolysosomes (M/E) into autophagosomes (AP) is also observed (black arrowheads) (**C**,**D**). Autophagosomes/autolysosomes are formed in the vicinity of the Golgi apparatus (GA), particularly in close proximity to the trans-Golgi network (**D**,**F**–**I**). Mitochondria (MIT) are often found in close proximity to autolysosome membranes (**C**–**I**). Macromitophagy is also evident (white arrowhead) (**C**).

**Figure 5:**
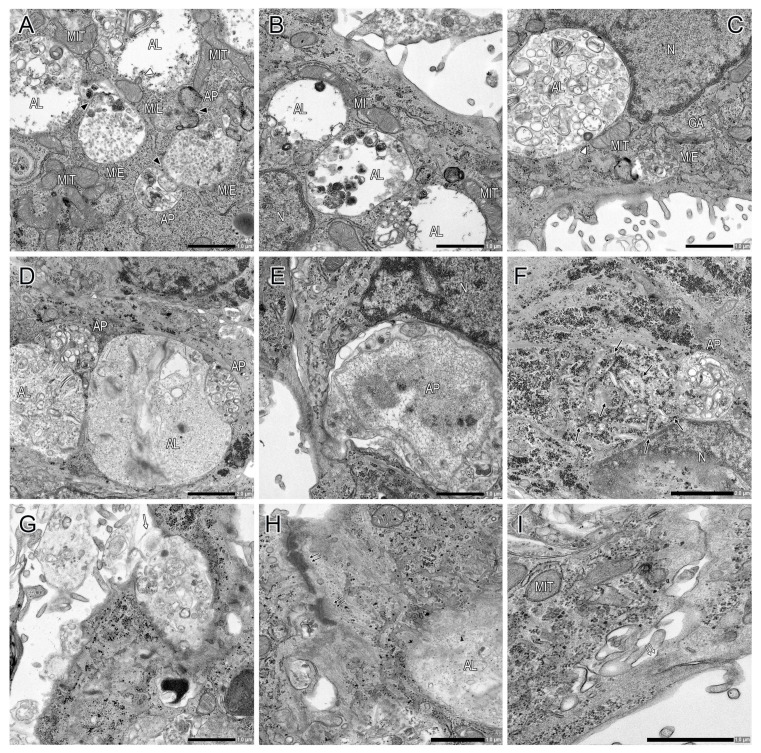
Ultrastructure of primary CTB cells at 72 h culture (part 2). (**A**–**D**) The main compartments of autophagy (phagophore (PP), autophagosome (AP), autolysosome (AL)), and MVE/endolysosome (M/E) are detected. The fusion of MVEs/endolysosomes (M/E) into autophagosomes (AP)/autolysosomes (AL) was also observed (black arrowheads) (**A**). Autophagosomes and autolysosomes undergo fusion, resulting in the formation of larger structures (open arrowheads) (**D**). Mitochondria (MIT) are often found in close proximity to autolysosome membranes (**A**–**C**). The fusion of mitochondria-derived vesicles with autolysosomes (microautophagy) is visible (white arrowheads) (**A**,**C**). (**E**) A nucleus is sequestered within an autophagosome. (**F**) In glycogen aggregation areas, many glycogen granules form autophagosomes (black arrows), which subsequently coalesce to form a large autophagosome (AP). (**G**) Extracellular secretion of an autophagy vesicle is detected (white arrow). (**H**) The remnants of desmosomes are present within cells (double black arrows). (**I**) A subset of CTB cells is in the process of CTB differentiation into syncytial cells, with cell protrusions undergoing fusion (double white arrows).

After a 6 h culture, CTB cells aggregated, and some of them overlapped each other (Figure 2A,D). Note that there was scant evidence of CTB differentiation into syncytial cells. CTB cells had a cuboidal and polygonal shape. They had ovoid euchromatic nuclei with the thin peripheral rim of heterochromatin and one or more prominent nucleoli (Figure 2A). In the CTB cells, the intracellular organelles present in epithelial-type cells were observed (Figure 3). Their cytoplasm contained rough endoplasmic reticulum, mitochondria, free ribosomes, polyribosomes, Golgi apparatus, lysosomes, endosomes that contain multivesicular endosomes (MVEs) and endolysosomes, lipid droplets, and cytoskeletal filaments (e.g., intermediate filaments), but very few secretory granules (Figure 3). Aggregates of glycogen were often present; nematosomes were occasionally seen (Figure 3L) [19]. Desmosomes and primitive adherens junction-like structures (slight thickenings of cell membranes) were visible at cell–cell interfaces (Figure 3A). Microvilli with coated pits and vesicles were evident on the cell surface (Figure 3B). The main compartments of the multi-step process of autophagy, i.e., phagophore, autophagosome, and autolysosome, were present in CTB cells at this time point (Figure 3), but autophagosome and autolysosome were diminutive structures in comparison to those observed in syncytial cells at 72 h culture (Figure 4 and Figure 5). In addition, the presence of mitophagy, that is, mitochondrial degradation for major pathways of mitochondrial quality, was occasionally discernible (Figure 3I) [20,21,22].

Following a culture period of 72 h, CTB cells demonstrated to promote CTB differentiation into syncytial cells (Figure 2B,C,E). Electron microscopy visualized dynamic change in characteristics of intracellular organelle profiles in the syncytial cells; the presence of a significant number of gigantic autophagosomes/autolysosomes in the perinuclear area was identified (Figure 2, Figure 4 and Figure 5). In the syncytial cells, the multinucleated nuclei exhibited increased size, irregularity, and indentation compared to the CTB nuclei at 6 h culture (Figure 2); additionally, the peripheral chromatin demonstrated further condensation. Desmosomes were still identifiable at the cell–cell interfaces, in conjunction with the remnants of desmosomes within the cells (i.e., remnants of desmosomes after fusion) (Figure 5H). A subset of CTB cells were not fully fused but rather were in the process of CTB differentiation into syncytial cells, with some of the cell protrusions undergoing fusion (Figure 5I).

An increase in the number and size of autophagosomes/autolysosomes was noticeable (Figure 4A). The main compartments of the multi-step process of autophagy were readily detectable (Figure 4 and Figure 5). The fusion of MVEs/endolysosomes into autophagosomes/autolysosomes was also observed (Figure 4C,D and Figure 5A). Autophagosomes and autolysosomes underwent fusion, resulting in the formation of larger structures (Figure 5D). Interestingly, these autophagosomes/autolysosomes appeared to be formed in the vicinity of the Golgi apparatus, particularly in close proximity to the trans-Golgi network (Figure 4D,F–I). Mitochondria were often found in close proximity to autolysosome membranes (Figure 4C–I and Figure 5A–C); moreover, an observation was made regarding the fusion of mitochondria-derived vesicles with autolysosomes (Figure 5A,C). Additionally, some nuclei underwent autophagy (Figure 5E), and in glycogen aggregation areas, many glycogen granules formed autophagosomes, which subsequently coalesced to form large autophagosomes (Figure 5F). Macromitophagy, which involves the sequestration of mitochondria within double-membrane vesicles, i.e., autophagosomes, was observed (Figure 4C). Although extracellular secretion of autophagy vesicles (e.g., autolysosome-like vesicles) was detected (Figure 5G), its frequency of occurrence was likely limited. The ultrastructure of autophagosomes was clearly demonstrated; however, capturing images of the fusion of typical lysosomes and autophagosomes proved challenging.

## 3. Discussion

Villous surface-covering STB is of the utmost importance, as it fulfills a variety of functions that are vital to both the growth of the fetus and the maintenance of the pregnancy. The STB and its progenitor cell, CTB cells, are specialized epithelial cells that are unique to pregnancy. Abnormalities in these cells are closely linked to the pathology of pregnancy complications, e.g., preeclampsia [23]. Due to ethical considerations, in vitro analysis of trophoblast cells isolated from human placentas is vital to elucidate the molecular mechanisms underlying placental development and function. Furthermore, recent advancements in the field, including in vitro trophoblast cell models derived from diverse stem cell sources and three-dimensional culturing systems (i.e., trophoblast organoid and placenta-on-a-chip model), have enabled the facilitation of research on human trophoblast biology as in vitro models that more closely resemble in vivo conditions [24]. In this study, we isolated term villous CTB cells using the most up-to-date isolation method for isolating pure CTB cells from human term placenta [11] and investigated the ultrastructure of CTB differentiation into syncytial cells by means of transmission electron microscopy. Electron microscopy analysis demonstrated upregulation of autophagy and visualized autophagic profiles during differentiation into syncytial cells. Although electron microscopy analysis has been exclusively an accompaniment to functional analysis in previous reports [12,13,14], to the best of our knowledge, this is the first report of detailed ultrastructural investigation of autophagy flux of primary human term trophoblast cells.

Autophagy is a process that occurs within cells whereby cytoplasmic materials are degraded and recycled [25]. This process plays a fundamental role in cellular physiology, helping to maintain nutrient homeostasis and quality control of organelles at the cell, tissue, and organ levels [26]. The autophagic pathways are categorized into three types: macroautophagy, microautophagy, and chaperone-mediated autophagy [27,28]. Macroautophagy commences with phagophore and generates double-membrane structured autophagosome formation, which encapsulates the target cytosolic components; autophagosomes fuse with lysosomes, forming autolysosomes where contents are degraded by lysosomal hydrolases [29,30]. Microautophagy is the membrane internalization of the lysosomal or endosomal membrane to generate intraluminal vesicles containing cytosolic materials that are eventually broken down in lysosomes [22].

In the present study, we revealed the ultrastructural features of syncytial trophoblast cells, which exhibited a substantial number of remarkably large autophagosomes/autolysosomes (Figure 2, Figure 4 and Figure 5). The mechanisms underlying the formation and sustenance of large autophagosome/autolysosome structures in the syncytial cells remain to be elucidated. However, one hypothesis posits the potential involvement of autophagic lysosome reformation (ALR) [31,32]. Autophagosomal and autolysosomal membrane proteins are subject to recycling via the process of autophagosome and autolysosome reformation, which is commonly referred to as ALR. These reformations induce bulk transport of their contents as membrane fragments to lysosomes, resulting in the formation of mature functional lysosomes to sustain autophagy function. ALR inhibition leads to the result of enlarged and long-lasting autolysosomes [33]. We also found gigantic fused autophagosomes/autolysosomes in close proximity to the trans-Golgi network in multinucleated syncytial cells (Figure 4D,F–I). Autophagosomes are generally formed throughout the cytoplasm [34]. However, under conditions of nutrient starvation, there is a perinuclear accumulation of lysosomes; this phenomenon has been hypothesized to promote the initiation of autophagy and the subsequent fusion of autophagosomes with lysosomes [35]. In that context, the results obtained in this study imply a propensity for the initiation of autophagy, as well as the subsequent autophagosome-lysosome fusion.

Extracellular secretion of autophagy vesicles was observed in syncytial trophoblast cells (Figure 5G). Autophagy and endocytosis pathways crosstalk at several stages throughout intracellular vesicle biogenesis, transport, fusion, degradation, and recycling [36]. MVEs are detached from early endosomes and further mature into endolysosomes and late endosomes [37,38,39]. MVEs can also fuse with the plasma membrane and release their intraluminal vesicles as exosomes [40]. In the human placenta, villous trophoblast exosomes are continuously synthesized and released into the maternal circulation throughout the gestational period [41]. Trophoblast exosomes promote fetomaternal immunotolerance [42,43] and antiviral defense [12,44]. Recent studies have demonstrated the presence of heterogeneous extracellular vesicle populations, including exosome types [40,45]. In addition to canonical endosome-derived MVEs, non-canonical MVEs have been reported to originate from autolysosomes, autophagosomes, and mitochondria [40]. The revaluation of cargo delivery vehicles for trophoblast-associated cargos that are secreted through autophagy and endocytosis pathways using in vitro STB models remains to be elucidated.

It has been reported that the STB exhibits elevated levels of basal autophagy activity [46,47]. This intrinsic autophagy has been shown to play a crucial role in antiviral defense [12] and the maintenance of placental homeostasis [48]. Autophagy is also involved in pregnancy complications (e.g., preeclampsia and fetal growth restriction) [49]; autophagy and lysosomal biogenesis are impaired in preeclampsia patients [14]. Primary CTB cells also exhibit high levels of basal autophagy activity [12,50]. Chen et al. reported autophagy of primary term CTB cells and its upregulation in response to hypoxic stress [50]. Interestingly, Furuta et al. reported the requirement of autophagy for the initiation of syncytialization and gradual decrease of autophagy during syncytialization in trophoblast cells, although a trophoblastic cell line BeWo was employed for most autophagy flux analyses [51]. They also showed an increase in LC3-II expression on day 3, but not day 5, in culturing primary term trophoblast cells. In this study, the presence of high autophagy flux at the electron microscopic level in syncytial trophoblast cells on day 3 culture may suggest the visualization of the early stages of syncytialization. Given the ability of isolated pure CTB cells to culture as a model of the STB for up to 96–120 h [11], the investigation of ultrastructural alterations of the dynamic process of autophagy under conditions of stress, such as hypoxia and starvation, and during extended periods of incubation, promises to be a fascinating avenue of research.

This investigation is constrained by limitations. It is impossible to examine the dynamic process of autophagy using a solitary method; however, the present study concentrates on the electron microscopy analysis of isolated CTB cells, for which detailed autophagy ultrastructures have not been reported. There are as yet unresolved questions regarding the identity and composition of autophagy and endosome compartments and their crosstalk in the STB using primary trophoblast cells. Multimodal imaging techniques (e.g., high-resolution live-cell imaging and correlative light and electron microscopy) in conjunction with molecular cell biology approaches will undoubtedly provide further insight into trophoblast-associated autophagy [52].

## 4. Materials and Methods

### 4.1. Isolation and Culture of Primary Human Term Cytotrophoblast (CTB) Cells

Human placentas from pregnant women who provided informed consent were obtained using protocols approved by the Nippon Medical School Ethics Committee (Tokyo, Japan, nos. A-2021-060 [1 July 2021] and M-2022-053 [23 August 2022]) and the Jichi Medical University Ethics Committee (Tochigi, Japan, nos. 21-015 [16 November 2021] and 22-153 [26 April 2023]).

CTB cells were isolated from human term placentas (at 37–38 weeks of gestation, n = 5) using the improved isolation method by Motomura et al. [11]. Briefly, placental villous tissues were minced and digested with a digestion buffer (1× Hank’s balanced salt solution [HBSS; cat. no. 14185-052, Gibco, Grand Island, NY, USA] with 25 mM HEPES [cat. no. H0887-100ML, Sigma Chemical Corp., St. Louis, MO, USA], containing 0.25% [2500 UPS U/mL] Trypsin [cat. no. 15090-046, Gibco], 2 mg/mL [2 USP U/mL] Dispase II [cat. no. 04942078001, Roche Diagnostics GmbH, Mannheim, Germany], and 152 μg/mL [>300 U/mL] DNase I [cat. no. 10104159001, Roche Diagnostics GmbH]) in a 37 °C water bath (PERSONAL-11, TITEC Corp., Saitama, Japan) at 150 rpm rotation speed for 20 min. The enzyme digestion was repeated three times. Cells obtained by the enzymatic digestion were filtered through a 100 μm nylon cell strainer (cat. no. 352360, Falcon, Durham, NC, USA) and then fractionated by Percoll density gradient centrifugation at 1200× *g* for 20 min at 22 °C in a swinging bucket rotor without an accelerator and a brake (S300TR, KUBOTA, Tokyo, Japan); a Percoll density gradient was prepared by layering 3 mL of Percoll dilutions [layers from the bottom (70%) to the top (5%) at 5% intervals; cat. no. 17544502, Cytiva, Tokyo, Japan). The white band above the 50% Percoll layer in which CTB cells were enriched were collected. Remnants contaminating non-CTB, HLA class I-positive cells in the enriched CTB cell collection were extensively removed by the magnetic-activated cell sorting (MACS) negative selection technique (Miltenyi Biotec, Bergisch Gladbach, Germany) employing an anti-HLA ABC mouse monoclonal antibody (clone W6/32, cat. no. 16-9983-85, Invitrogen, Waltham, MA, USA) and Anti-Mouse IgG MicroBeads (cat. no. 130-048-401, Miltenyi Biotec). Cell number and cell viability of HLA class I-negative cells (i.e., CTB cells) were 3.0–4.0 × 10^8^ cells and >95%, respectively.

Without cell storage at −80 °C, isolated CTB cells were plated at a cell density of 1.3 × 10^6^ cells/cm^2^ in Iscove’s modified Dulbecco’s medium (IMDM) with phenol red, L-glutamine free (cat. no. I3390-500ML, Sigma Chemical Corp.) supplemented with 10% FBS (cat. no. FB-1290/500, Biosera, Nuaille, France), 2 mM L-glutamine (cat. no. 25030-081, Gibco), and 100 IU/mL penicillin/streptomycin/amphotericin B (cat. no. 161-23181, FUJIFILM Wako Pure Chemical Corp., Osaka, Japan) and cultured for 6–72 h at 37 °C under 5% CO_2_ in 96-well plates (3.8 × 10^5^ cells/100 µL/well; cat. no. 167008, Thermo Fisher Scientific, Waltham, MA, USA) for cell viability assay, 24-well plates (2.5 × 10^6^ cells/500 µL/well; cat. no. 142475, Thermo Fisher Scientific) for immunofluorescence analysis, and 35-mm plastic culture dishes (1.1 × 10^7^ cells/2 mL/dish; cat. no. 150460, Thermo Fisher Scientific) for transmission electron microscopy analysis.

### 4.2. Immunofluorescence

The syncytium formation was visualized by the immunofluorescence of CDH1, which is localized to the cell–cell contact sites on the cell surface of primary CTB cells [53]. The Immunofluorescence of KRT7, a marker of trophoblast cells, was simultaneously performed to verify the purity of primary CTB preparations [54,55]. Immunofluorescence was performed as described previously [56]. Briefly, isolated CTB cells were cultured on 0.01% poly-L-lysine hydrobromide (cat. no. P2636, Sigma Chemical)-coated round glass coverslips (12 mm in diameter; cat. no. C012001, Matsunami, Osaka, Japan) in 24-well plates. The cells were fixed with 4% paraformaldehyde (cat. no. P001, TAAB, Aldermaston, UK) in phosphate-buffered saline (PBS), pH 7.4, for 2 h at 22 °C and permeabilization was performed with 0.2% Triton X-100 (cat. no. X100-100ML, Sigma Chemical Corp.). After blocking buffer treatment (FBS containing 1% bovine serum albumin [cat. no. A7906, Sigma Chemical Corp.] and 5% normal goat serum [cat. no. G9023, Sigma Chemical Corp.]) for 30 min at 22 °C, samples were incubated for 30 min at 37 °C with primary antibodies and subsequently for 30 min at 37 °C with Alexa-Fluor 488- and 594-conjugated secondary antibodies (5–10 µg/mL) (Molecular Probes, Eugene, OR, USA). Control samples received the same treatment, with the exception that the primary antibodies were either omitted or replaced with non-immune IgG (mouse IgG1κ [cat. no. X0931, Dako, Glostrup, Denmark] and rabbit IgG [cat. no. ab27478, Abcam, Cambridge, UK]). The samples were then counterstained with 4,’6-diamidino-2-phenylindole dihydrochloride (DAPI; cat. no. D1306, Thermo Fisher Scientific) and mounted in ProLong Diamond Antifade Mountant (cat. no. P36965, Invitrogen). The samples were examined using a laser scanning AiryScan-LSM980 Axio Observer confocal microscope (Carl Zeiss, Jena, Germany); digital images were captured with ZEN Microscopy software (version 3.8.99.00000, Carl Zeiss). Confocal images were compiled using Adobe Photoshop software (version 26.3.0, Adobe Systems, San Jose, CA, USA).

Antibodies used for immunofluorescence were as follows: rabbit anti-CDH1 (cat. no. sc-7870, Santa Cruz Biotechnology, Santa Cruz, CA, USA), mouse anti-KRT7 (cat. no. M7018, Dako, Agilent Technologies, Santa Clara, CA, USA), and Alexa-Fluor 488- and 594-conjugated secondary antibodies (cat. nos. A32731 and A11032, Molecular Probes).

### 4.3. Transmission Electron Microscopy

After culture of isolated CTB cells for 6 h and 72 h in 35-mm dishes, cells were fixed in 2% paraformaldehyde and 2.5% glutaraldehyde (cat. no. G004, TAAB, Aldermaston, UK) in 0.1 M sodium cacodylate buffer, pH 7.4, containing 0.05% CaCl_2_ for more than 5 h at 22 °C and then postfixed in 2% osmium tetroxide (cat. no. 1.24505.0001, Merck KGaA, Darmstadt, Germany) in 0.1 M sodium cacodylate buffer, pH 7.4, containing 1.6% potassium ferrocyanide (cat. no. 163-03741, FUJIFILM Wako Pure Chemical Corp.) for 60 min at 4 °C. Samples were then dehydrated in graded ethanol series and were subsequently embedded in Quetol-812 (Nissin EM, Tokyo, Japan). Ultrathin sections were stained with uranyl acetate and lead citrate and examined in a JEM-1400Plus electron microscope (JEOL, Tokyo, Japan) operated at 80 kV. Micrographs were compiled using Adobe Photoshop software.

## 5. Conclusions

In conclusion, we isolated human term villous CTB cells using the most up-to-date isolation method for isolating pure CTB cells from human term placenta and investigated the ultrastructure of CTB differentiation into syncytial cells by means of transmission electron microscopy. The initial 6 h culture resulted in CTB cell aggregation; however, the majority of CTB cells did not differentiate into syncytial cells. In contrast, after 72 h, CTB cells exhibited a promotion of differentiation into syncytial cells. Electron microscopy analysis demonstrated the upregulation of autophagy and visualized unique autophagic ultrastructural profiles during differentiation into the syncytial cells, which exhibited perinuclear accumulation of extremely large autophagosomes/autolysosomes. Ultrastructural elucidation in the dynamic process of autophagy and endocytosis in trophoblast cells and its significance in terms of biological functions is likely to provide exciting avenues for the reproductive biology of the STB.

## Data Availability

All data generated or analyzed during this study are included in this published article.

## Abbreviations

STB	Syncytiotrophoblast
CTB	Cytotrophoblast
CDH1	Cadherin 1
KRT7	Keratin7
MVE	Multivesicular endosome
ALR	Autophagic lysosome reformation
